# Structure, function, and substrates of Clp AAA+ protease systems in cyanobacteria, plastids, and apicoplasts: A comparative analysis

**DOI:** 10.1016/j.jbc.2021.100338

**Published:** 2021-01-23

**Authors:** Imen Bouchnak, Klaas J. van Wijk

**Affiliations:** Section of Plant Biology, School of Integrative Plant Sciences (SIPS), Cornell University, Ithaca, New York, USA

**Keywords:** Proteases, plastids, apicoplasts, chloroplasts, AAA+ proteins, Clp protease, N-degrons, protease adaptors, proteostasis, AAA+, ATPases Associated with diverse cellular Activities, Clp, caseinolytic protease, cpUPR, chloroplast Protein Unfolding Response, DXS, deoxyxylulose 5-phosphate synthase, GBP, GluTR-binding protein, GluTR, glutamyl t-RNA reductase, GUN, Genomes Uncoupled 1, IPP, isopentenyl pyrophosphate, PTM, post-translational modification, UPR, unfolded protein response

## Abstract

ATPases Associated with diverse cellular Activities (AAA+) are a superfamily of proteins that typically assemble into hexameric rings. These proteins contain AAA+ domains with two canonical motifs (Walker A and B) that bind and hydrolyze ATP, allowing them to perform a wide variety of different functions. For example, AAA+ proteins play a prominent role in cellular proteostasis by controlling biogenesis, folding, trafficking, and degradation of proteins present within the cell. Several central proteolytic systems (*e.g.*, Clp, Deg, FtsH, Lon, 26S proteasome) use AAA+ domains or AAA+ proteins to unfold protein substrates (using energy from ATP hydrolysis) to make them accessible for degradation. This allows AAA+ protease systems to degrade aggregates and large proteins, as well as smaller proteins, and feed them as linearized molecules into a protease chamber. This review provides an up-to-date and a comparative overview of the essential Clp AAA+ protease systems in Cyanobacteria (*e.g*., *Synechocystis* spp), plastids of photosynthetic eukaryotes (*e.g.*, *Arabidopsis*, *Chlamydomonas*), and apicoplasts in the nonphotosynthetic apicomplexan pathogen *Plasmodium falciparum*. Recent progress and breakthroughs in identifying Clp protease structures, substrates, substrate adaptors (*e.g*., NblA/B, ClpS, ClpF), and degrons are highlighted. We comment on the physiological importance of Clp activity, including plastid biogenesis, proteostasis, the chloroplast Protein Unfolding Response, and metabolism, across these diverse lineages. Outstanding questions as well as research opportunities and priorities to better understand the essential role of Clp systems in cellular proteostasis are discussed.

## Proteostasis

Protein homeostasis or 'proteostasis' is the process that regulates proteins within the cell to maintain the health of both the cellular proteome and the organism itself. Proteostasis requires integration of protein synthesis, sorting, folding, maturation, and degradation. Proteases play a very central role in proteostasis because they contribute in many different ways, including (i) removal of presequences that are required for intracellular sorting but must be removed after sorting is completed, (ii) removal of N-terminal methionines, (iii) additional N- or C-terminal cleavages for maturation and stabilization and possibly activation of proteins, (iv) removal of miss-folded, damaged, or aggregated proteins, (v) removal of unwanted proteins in response to environmental or developmental transitions, and (vi) metabolism of proteins as alternate respiratory substrates in times of stress. Proteolysis is therefore central to proteostasis and fitness of the cell. A key challenge in understanding cellular proteostasis is determining how substrates are recognized by proteases and how the activity of proteases is regulated. This recognition typically involves modification of proteins resulting in the formation of a protein degradation signal, commonly assigned as a degron (1). Degrons can be the unfolded domain of a protein or can involve one or more post-translational modifications (PTMs). These degrons can be located at the N-terminus or C-terminus of a protein but also anywhere in between. Much research has focused on N-terminal degrons and the so-called N-end rule degradation pathway, initially discovered in yeast (2) but subsequently shown to be relevant for other eukaryotes and prokaryotes (3, 4). Mechanisms through which proteases recognize their substrates are hard to predict and therefore require experimentation.

## Protease systems with AAA+ proteins

There are many types of proteases, and a universal classification system for peptidases has been developed in which each protein is assigned to a family based on similarities in amino acid sequence (5). Many prominent and well-studied proteases are members of the protein superfamily ATPases Associated with diverse cellular Activities (AAA+). AAA+ proteins perform a wide variety of different functions essential to cell physiology, including control of protein homeostasis, DNA replication, recombination, chromatin remodeling, ribosomal RNA processing, molecular targeting, organelle biogenesis, and membrane fusion (6, 7). They typically form hexameric complexes and act as motors to remodel other proteins. Several central and prominent proteolytic systems use AAA+ proteins to unfold protein substrates to make them accessible for degradation. This allows AAA+ protease systems to degrade aggregates and large proteins, as well as smaller proteins, by feeding them as linearized molecules into a protease chamber protected from the cellular environment. Examples of AAA+ protease systems are the 26S proteasome (8) and the caseinolytic protease (Clp), FtsH, Deg, and Lon protease systems (9, 10). AAA+ proteins share an AAA+ domain with two canonical motifs (Walker A and B) required for ATP binding and hydrolysis (6, 7). The P-loop of the Walker A motif directly interacts with the phosphates of ATP, whereas a specific lysine residue within this P-loop (in the consensus sequence GXXXXGK[T/S]) is crucial, and its mutation prohibits nucleotide binding and inactivates the AAA+ protein (6, 7). The Walker B motif also interacts with the adenine nucleotide, and acidic residues (D and E) within a hydrophobic sequence (hhhhDE) are crucial for ATPase activity. The aspartate residue (D) recruits Mg^2+^ required for ATP hydrolysis, whereas the glutamate residue (E) is essential to activate water for the hydrolysis reaction. Mutations in the Walker B motif can be used as effective tools to trap substrates that will bind but cannot be released, as reviewed (11).

## Plastids are essential organelles in algae, plants, and most apicomplexan

**Figure 1:**
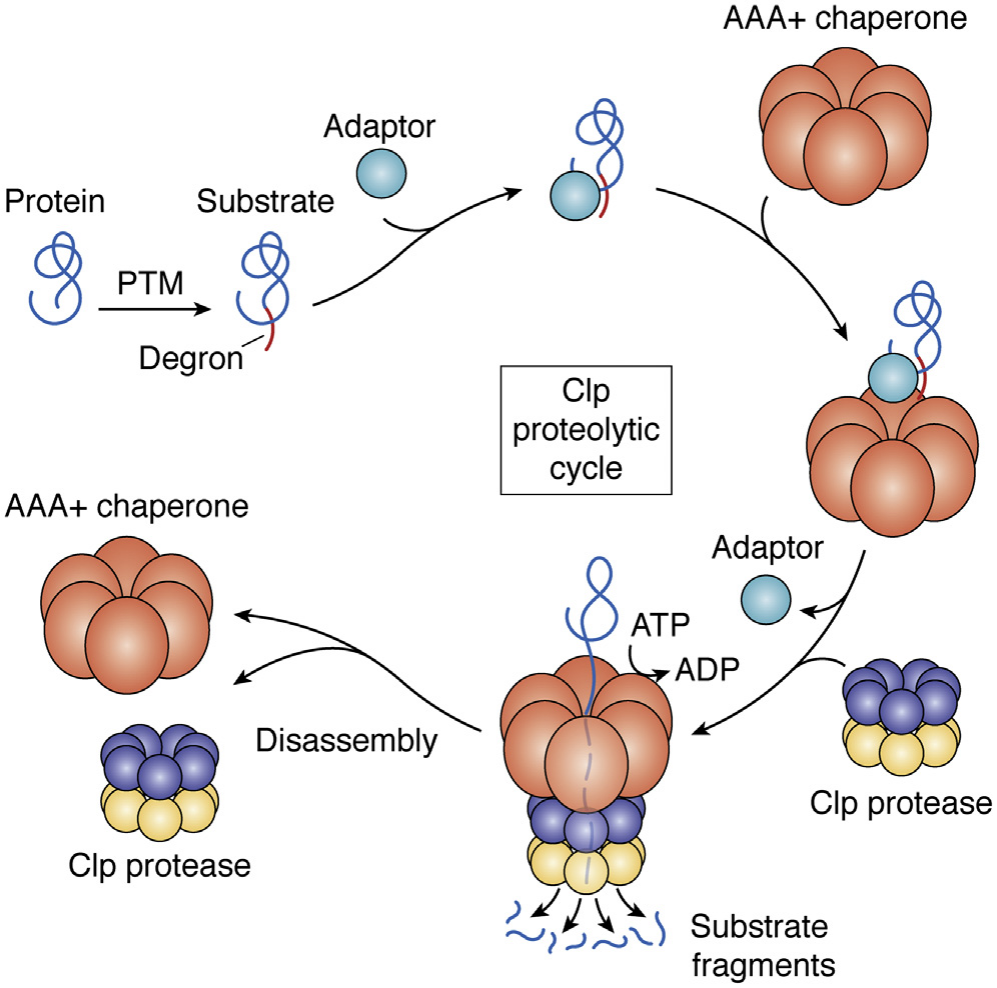
**Evolution of the Clp protease system from cyanobacteria to chloroplasts in higher plants to apicoplasts in apicomplexan pathogens.** Primary plastids evolved from cyanobacteria that were engulfed by a eukaryotic cell (primary endosymbiosis). Apicoplasts evolved from a plastid containing eukaryote that was engulfed by another nonphotosynthetic eukaryotic cell (secondary endosymbiosis). The so far studied cyanobacterial Clp systems consist of (1) ClpS1 and ClpS2 and NblA as the adaptor proteins and (2) ClpC and ClpX (*yellow* and *blue*) as the chaperone subunits for ClpP3R and ClpP1P2 heterotetradecameric protease core complexes, respectively. The plant plastid Clp system consists of (1) ClpS1 and ClpF as the adaptor proteins, (2) ClpC1, C2, and D (*yellow*) as the chaperone subunits, (3) heterotetradecameric protease core complexes consisting of the so-called R-ring with ClpP1, R1-4, and the P-ring with ClpP3-6 subunits, (4) ClpT1 and ClpT2 (*pink*) as the accessory proteins associated with the P-ring. The apicoplast Clp system consists of (1) ClpS as the adaptor protein, (2) ClpC (*yellow*) as the chaperone, and tetradecameric ClpP3 (*gray*) and ClpR (*black*) likely assembled as a single heterotetradecameric core complex. A list of Clp subunits and their functional role for the different biological systems is provided in Table 1. Clp, caseinolytic protease.

Plastids are essential organelles in photosynthetic eukaryotes (algae and plants) and most apicomplexan. Plastids in algae and plants are best known for their role in photosynthesis, but they also carry out the biosynthesis of many primary and secondary metabolites such as fatty acids, amino acids, vitamins, nucleotides, tetrapyrroles, and hormones (12, 13, 14). Plastids in photosynthetic organisms undergo various developmental transitions and adaptations, from nonphotosynthetic plastids in roots to photosynthetic chloroplasts in green tissues (13). Each plastid type contains its specific proteome through the coordinated actions of the proteostasis network, involving transcription, translation, protein folding, and degradation machineries. The remodeling and stability of these proteomes during plastid differentiation and adaptation must occur through selective protein synthesis and proteolysis. Understanding the proteolytic hierarchies and degrons is therefore essential to understand plastid differentiation, adaptation, and function (15, 16, 17). Apicoplasts are nonphotosynthetic plastids, derived from secondary endosymbiosis (Fig. 1), found in most Apicomplexa, including *Toxoplasma gondii*, *Plasmodium falciparum*, and other *Plasmodium* spp., but not in others such as Cryptosporidium (18). Apicoplasts contribute to amino acid, fatty acid, and isoprenoid metabolism quite comparable with the functions of plastids in plants and algae, but they are never photosynthetic.

## AAA+ protease systems in cyanobacteria, plastids, and apicoplasts

The Clp, FtsH, Deg, and Lon AAA+ protease systems in plastids and apicoplasts are derived from cyanobacterial progenitors (Fig. 1). However, after endosymbiosis, these protease systems diversified and expanded with additional components, as reviewed (15). Examples are the dramatic increase in the number of FtsH, ClpP, and ClpR proteins, as well as novel auxiliary proteins such as ClpT in plant and algal plastids (Table 1). Over the last decade, there has been substantial progress in identifying structures, functional roles, and substrates of these AAA+ protease systems in cyanobacteria, plastid, and apicoplasts, as summarized in several general reviews on plastid proteases (15, 16, 19) as well as specific reviews for the Clp system (20, 21), FtsH (22), Deg (23), and Lon (24). Each of these AAA+ proteolytic systems is important to biogenesis and function of cyanobacteria and plastids. Loss of function of these proteases can result in lethality in the case of the Clp system as will be discussed further in this review, whereas loss of other AAA+ proteases generally results in milder phenotypes that, however, can affect biogenesis or fitness under specific stress conditions such as heat or excess light.

**Table 1:** Clp proteins in cyanobacteria, plants, algae, and apicoplasts

Functional components of the Clp system	Clp subunit type	Cyanobacteria^a^	Plant plastids^a^	Algal plastids^a^	Apicoplasts^a^
AAA+ chaperones	ClpC^b^	×	×	x	×
	ClpD^c^		×		
	ClpX^d^	×			
Catalytically active protease	ClpP^e^	×	×	×	×
Catalytically inactive protease	ClpR^f^	×	×	×	×
Auxiliary	ClpT^g^		×	×	
Recognins/adaptors	ClpS^h^	×	×	×	×
	ClpF^i^		×		
	NblA^j^	×			

AAA+, ATPases Associated with diverse cellular Activities; Clp, caseinolytic protease.

a The analysis of the Clp system is mostly based on research in a limited set of species. For cyanobacteria, these are *Synechocystis elongatus PCC 7942*, *Synechocystis* sp. *PCC803*, and *Anabaena PCC 7120*. For algae, this is *Chlamydomonas reinhardtii*. For plants, this is mostly *Arabidopsis thaliana* and to a lesser extent also tobacco, tomato, maize, and rice. For apicomplexan, this is *Plasmodium falciparum*.

b Cyanobacteria and apicoplasts have one ClpC homolog, whereas *Arabidopsis* has two ClpC homologs (ClpC1 and ClpC2).

c ClpD is a chaperone specific to plants and lacks a UVR domain. Its expression is induced by drought and during senescence.

d ClpX is not found in plastids, but cyanobacteria have one ClpX homolog. It should be noted that mitochondria have one or more ClpX homologs. *Arabidopsis* has three ClpX homologs.

e ClpP is the serine-type proteolytic subunit with a conserved catalytic triad. Cyanobacteria have three ClpP subunits (P1, P2, and P3). *Arabidopsis* has six ClpP subunits (P1-P6), of which ClpP1 is plastid-encoded and the others are nuclear-encoded. Only ClpP2 is located in mitochondria. Chlamydomonas has three plastid-localized Clp subunits (P1, P4, and P5), one of which is plastid-encoded (ClpP1). Apicoplasts have just one plastid-localized Clp subunit.

f ClpR is a homolog of ClpP but lacks the catalytic triad and is therefore not catalytically active. Cyanobacteria have one ClpR subunit, *Chlamydomonas* and *Arabidopsis* have each four ClpR subunits (R1-R4), and apicoplasts have one ClpR subunit.

g ClpT is unique to algae and higher plants; however, they show very low sequence similarity. ClpT subunits associate with the ClpP ring and likely play a role in the activation of the Clp core or perhaps substrate delivery independent of Clp chaperones. *Arabidopsis* and *Chlamydomonas* each have two ClpT subunits.

h ClpS is a highly conserved adaptor or recognin acting specifically in recruiting substrates with an N-terminal degron. Cyanobacteria have ClpS1 and ClpS2 homologs, whereas as plastids in algae and plants only have ClpS1 homologs (typically just one). Apicoplasts have one ClpS homolog.

i ClpF is an adaptor function (or perhaps also anti-adaptor) and is unique to higher plants. It associates with both ClpS and ClpC homologs and it has both a UVR and YCCV domain.

j Cyanobacterial NblA (nonbleaching protein A) is a specialized ClpC adaptor to deliver phycobilisome proteins for degradation. Some species have two NblA homologs.

The FtsH systems are bound to membranes and are important in the removal of membrane proteins within the membranes where these FtsH proteases reside. The Clp, Lon, and Deg protease systems are soluble protein complexes degrading, however, not only soluble proteins but likely also membrane proteins in particular after they have been partially released from the membrane by FtsH or other membrane-bound proteases. Substrate recognition and delivery mechanisms, as well as degrons, remain poorly understood for these systems; arguably, understanding the rules for substrate recognition should be the top priority for studying protein degradation by these AAA+ protease systems. These AAA+ protease system functions as a protease network together with additional proteases (25), similarly as postulated for other cellular proteases (26) and proteostasis components including chaperones (27). Not much is known to what extent the different proteases in a cell or subcellular compartment act in parallel or in series to degrade proteins to small peptides or individual amino acids. mRNA-based coexpression analysis and genetic interaction studies using loss-of-function protease mutant lines can provide insight into these networks for plastids and mitochondria as we discussed previously (25).

In the present review, we provide an update on the Clp AAA+ protease systems found in cyanobacteria, plastids of algae and plants, as well as apicoplasts in the nonphotosynthetic apicomplexan pathogen *P. falciparum*, the causal agent of malaria. We focus on the Clp system rather than the FtsH, Lon, or Deg proteases because of the high number of recent publications and findings for the Clp system. Comparison of Clp systems across cyanobacteria and plastid organelles allows the study of the diversification and adaptation of these AAA+ protease systems (21, 28). We compare and discuss progress and challenges in identifying cyanobacterial, plastid, and apicoplast Clp protease structures, substrates, substrate adaptors (*e.g.*, NblA, ClpS), and their degrons.

## The cycle of substrate selection and degradation by the Clp system

**Figure 2:**
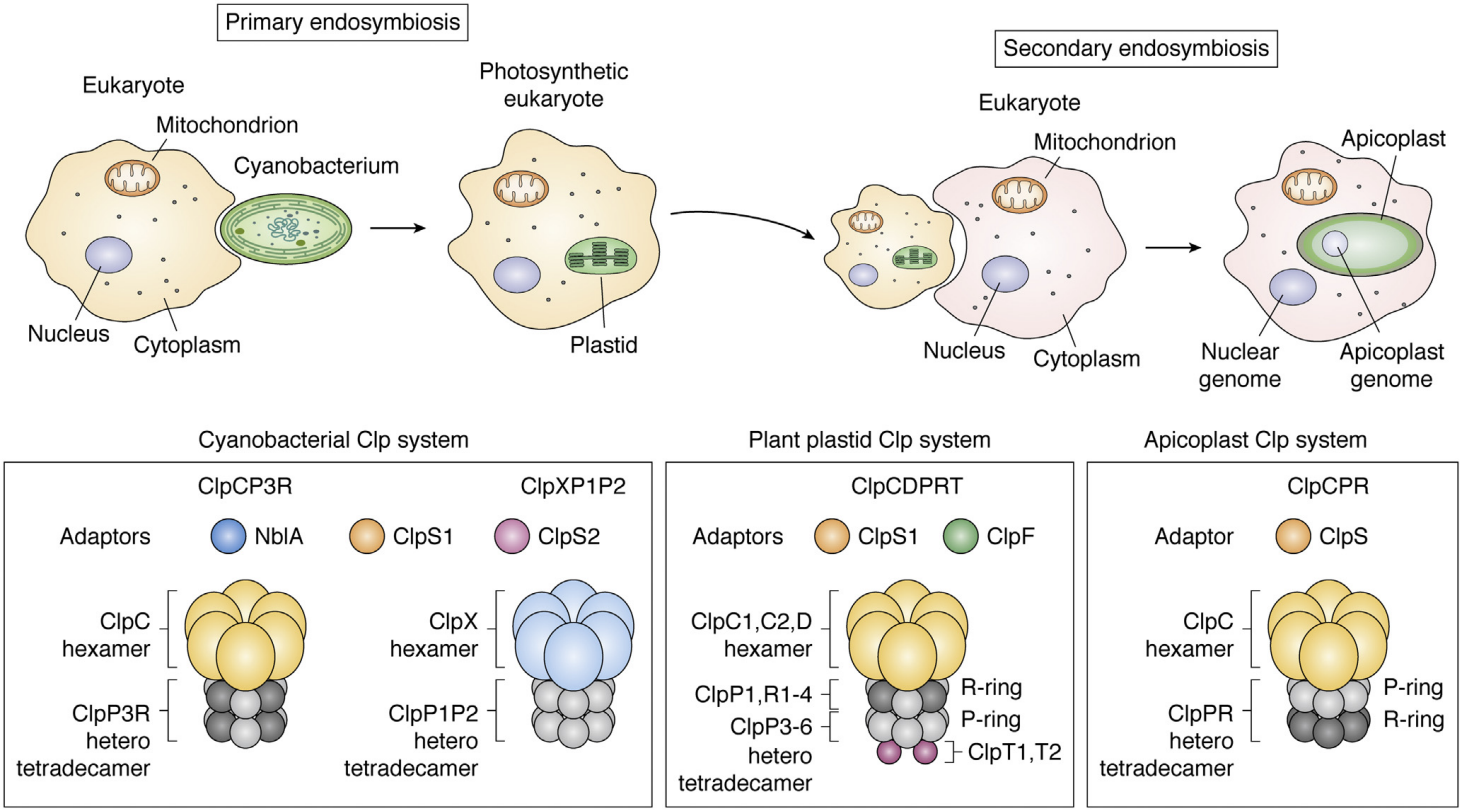
**Working model of the Clp proteolytic cycle and the Clp components.** Proteins become substrates after undergoing a post-translational modification (PTM) that must result in the generation of a substrate recognition signal, a degron. The substrate is recruited to the Clp chaperone complex, possibly in dependence of an adaptor (or an adaptor complex). ATP-dependent unfolding and threading of the substrate through the central pore of the Clp hexamer follows, and the Clp protease core complex docks onto the hexameric Clp chaperone. Small substrate fragments (∼6–9 aa) are released from the Clp protease core through dynamic lateral pores, and once the substrate degradation is completed, the Clp chaperone–protease complex disassembles. Clp chaperones may accumulate as dimers when not engaged in the degradation cycle and the formation of the chaperone hexamer requires priming of the chaperone by adaptors and/or ATP, leading to the formation of the activate hexamer in ATP-bound state. Clp, caseinolytic protease.

Clp-dependent proteolysis is an ATP-dependent multistep-regulated process that involves one or more Clp chaperones assembled into hexamers and a Clp protease core complex consisting of two stacked heptameric rings forming a tetradecameric complex (Figs. 1 and 2; Table 1). Proteins become substrates after undergoing PTMs that must result in the generation of a degron. The degron-containing substrate is now recognized directly by the Clp chaperone(s), but this can also involve active recruitment by so-called adaptor proteins or recognins, or even other chaperones, for example, members of the Hsp70 family in case of misfolded or aggregated protein substrates (Fig. 2). Upon interaction of the substrate with the Clp chaperone, the ATP-dependent substrate unfolding process starts and the Clp protease core complex is recruited to the substrate–chaperone assembly. ATP binding and hydrolysis is required for substrate unfolding and mostly likely also for Clp chaperone oligomerization. In contrast, the actual proteolytic cleavage by the catalytic Clp protease core does not require ATP, but specific and dynamic interactions (docking) between the Clp chaperone hexamer and the Clp protease core are required for catalytic cleavage of peptidyl bonds in the substrate. Small substrate fragments (∼6–9 aa) are released from the Clp protease core through dynamic lateral pores and, once the substrate degradation is complete, the Clp chaperone–protease complex disassembles (Fig. 2). It should be noted that the Clp chaperones may accumulate as dimers (*e.g.*, in chloroplasts) when not engaged in the degradation cycle and that formation of the chaperone hexamer requires priming of the chaperone by adaptors and/or ATP, leading to the formation of the activate hexamer in the ATP-bound state (9, 29, 30).

## Organization, expression, coevolution, structures, and functions of Clp systems

The basic structure of the Clp machinery is generally conserved across cyanobacteria, plastids, and apicoplasts and consists of a cylinder-like protease core and an AAA+ chaperone ring complex (10, 21, 31) (Figs. 1 and 2; Table 1). The chaperone complex serves as a molecular gate that controls substrate access and recognizes, unfolds, and translocates protein substrates into the core cavity in an ATP-dependent manner. Recent cryoelectron microscopy studies of bacterial ClpAP, ClpXP1P2, and ClpXP assemblies have provided an unparalleled insight into the organization and dynamics of these supercomplexes from *Escherichia coli*, *Listeria monocytogenes*, and *Neisseria meningitis*, respectively (32, 33, 34). So far, no cryoelectron microscopy structures of Clp chaperone–protease assemblies have been resolved for cyanobacteria, plastids, or apicoplasts, but high-resolution structures for some of the individual Clp components have been determined by X-ray crystallography for these systems, as will be discussed below. Key differences across cyanobacteria, plastids, and apicoplasts are the number of different proteins that make up the tetradecameric protease core, the number of ClpC and ClpS homologs, and the presence of ClpX (only in cyanobacteria) or ClpD (only in higher plant plastids) or ClpT homologs (only in plastids of both algae and higher plants). In addition to ClpS homologs observed in all three ‘systems,’ just two additional substrate adaptors have been identified so far, namely ClpF for plastids and NblA for cyanobacteria. Based on identification of additional Clp adaptors in various nonphotosynthetic bacteria, as reviewed (9, 35), we speculate that cyanobacteria, plastids, and apicoplasts have additional adaptors that are likely to be diverse, as they probably evolved independently in each of the lineages. In the remainder of this section, we briefly summarize the organization, expression, coevolution, and structures of Clp systems across these systems and point out which components are essential.

### The Clp system in cyanobacteria

The cyanobacterial Clp system in *Synechocystis* species has mostly been studied in rod-shaped *S. elongatus* PCC 7942 and coccoid *Synechocystis* sp. PCC803. The Clp system in both species is comprised of a heterotetradecameric ClpP1P2 core (36) and an essential heterotetradecameric ClpP3R core, as reviewed (21). ClpX and ClpC are chaperones for the ClpP1P2 and ClpP3R cores, respectively (Fig. 1; Table 1). ClpP2 is on an operon with ClpX (37), and ClpP2 shows regulated (diel) polar cellular localization impacting its activity (36). ClpS1 and ClpS2 are adaptor proteins interacting with ClpC, but cyanobacteria contain an additional ClpC adaptor, NblA (some species have two NblA homologs), which is specialized to deliver phycobilisome (PBS) proteins for degradation (38, 39). The structures of NblA from *Anabaena PCC 7120*, *Thermosynechococcus vulcanus*, and *Synechococcus elongatu*s sp. PCC 794 have been determined by X-ray crystallography and were shown to form four-helix bundles of dimers (40, 41). The DNA damage repair protein UmuD was recently identified as an *in vivo* substrate for both ClpCP3R and ClpXP1P2. UmuD was found to have an unusual dual degron that is recognized by the ClpS homologs or by ClpXP (42) (ClpS homologs and N-degrons across cyanobacteria, plastids, and apicoplasts). ClpXP1P2 also plays an important function in regulating the circadian rhythm (36). The loss of ClpXP1P2 protease results in long-period circadian rhythms. Deletion of the ClpX chaperone, but not ClpP1/2, results in cell division defects in a manner that is dependent on the expression of a dusk-peaking factor (36). However, the Clp substrates that impact division and circadian rhythm are not yet known (36) (Discovery of Clp substrates and functions based on phenotypes, comparative proteomics and in vivo and in vitro affinity enrichment).

### The Clp system in plastids of algae and plants

The Clp protease in plastids of higher plants originates from its ancient cyanobacterial endosymbiont, but it increased in complexity since endosymbiosis (Fig. 1). Indeed, higher plant plastids and chloroplasts contain the most diversified Clp protease core compared with all other species. Forward and/or reverse genetics in *Arabidopsis*, maize, rice, and tobacco demonstrated the essential nature of the plastid Clp system, as reviewed (20, 21). Complete loss of ClpC chaperone or ClpPR protease capacity results in embryo lethality, whereas partial loss results in delayed growth and development and virescent leaves. The plastid Clp system in *Arabidopsis* consists of a hetero-oligomeric protease core comprising five proteolytically active subunits (ClpP1 and ClpP3-ClpP6) and four proteolytically inactive proteins (ClpR1-4), as well as two plant-specific accessory proteins (ClpT1,2), three AAA+ chaperones (ClpC1, ClpC2, and ClpD), and two adaptors (ClpS1 and ClpF) (43) (Fig. 1; Table 1). Plastids do not contain any ClpX homologs but instead are present in mitochondria. The ClpPR core is constructed from the heptameric P-ring containing the four ClpP subunits (P3: P4: P5: P6 = 1: 2: 3: 1) and the heptameric R-ring containing the four ClpR proteins and ClpP1 (P1: R1: R2: R3: R4 = 3: 1: 1: 1) (21). A recent study showed that there is a tight correlation between amino acid substitution rates in the plastid-encoded ClpP1 and the nuclear-encoded Clp subunits across a broad sampling of angiosperms, suggesting continuing selection on interactions within this complex (44). This is also consistent with the finding that ClpP5 (3 copies per Clp core) is crucial for Clp catalysis, whereas ClpP3 (one copy per core) plays an essential role in Clp structure even if its catalytic activity is dispensable (45). The ClpC1,2 and ClpD chaperones carry two ATPase domains and an IGF motif that is essential for binding to the Clp protease core complex. ClpC1,2 and likely also ClpD (but not experimentally demonstrated) are involved in gating the ClpPRT complexes. Whereas ClpC chaperones are found in cyanobacteria and a variety of bacteria (46), the ClpD chaperone is unique to plants and ClpD has only ∼45% sequence identity to ClpC proteins in plastids and cyanobacteria (21). Similar to the ClpC homologs, ClpD is a so-called class-1 HSP100 chaperone containing two conserved ATPase domains with all typical features (Walker A and B domains and the pore loops) per monomer, as well as the conserved IGF motif (21). However, ClpD neither has the R-motif nor the uvrB/C motif present in ClpC1,2 and ClpF. uvrB/C domains are generally believed to support protein–protein interactions https://prosite.expasy.org/PDOC50151. Removal of the R motif in cyanobacterial ClpC results in loss of interaction with the ClpP3/R core but enables binding of *E. coli* ClpP and subsequent proteolytic activity (47). It remains to be determined if ClpD (lacking this R motif) can interact with the plastid ClpPR complex. As we pointed out previously, the uvrB/C of ClpF was suggested to interact with the uvrB/C domain in the ClpC homolog (43), perhaps indicating that ClpF does not interact with ClpD. A recent X-ray structure showed that the N-domain of ClpD is structurally divergent from ClpC1, and on that basis, it was suggested that ClpD may have different substrate recognition mechanisms than ClpC homologs (48). Nuclear genes encoding for Clp subunits coexpress with each other, except ClpD which clearly coexpresses with proteins involved in drought stress and senescence (25). This sets ClpD apart as a chaperone with specific stress and developmental function (ClpD has also been named ERD1 for Early Drought Induced Protein 1), but substrates of ClpD are so far unknown. Identification of ClpD interactors and substrates should help identify ClpD function and perhaps explain its diversification from ClpC. Plants have one or two ClpS1 homologs that likely function to recruit chloroplast proteins with N-degrons to ClpC1,2 and perhaps also to ClpD, for degradation (4). ClpS1 interacts with the plant-specific ClpF protein that contains uvrB/C and YccV domains. We postulated that ClpS1 and ClpF form a binary complex for delivery of glutamyl t-RNA reductase (GluTR) and perhaps other substrates. The core domain of ClpS1 and the N-terminal/UVR domains of ClpF are important for ClpC1 interaction (43). Finally, plastids contain a fourth Clp chaperone, ClpB3 (49, 50), which also has two ATPase domains but lacks an IGF motif and is, therefore, unable to dock onto the Clp protease core (21). ClpB3 is a member of the well-studied group of ClpB unfoldases, which rescue aggregated proteins for reactivation rather than degradation in collaboration with HSP70 proteins (51, 52) (Other substrate adaptors and delivery systems).

### The Clp system in apicoplasts

**Figure 3:**
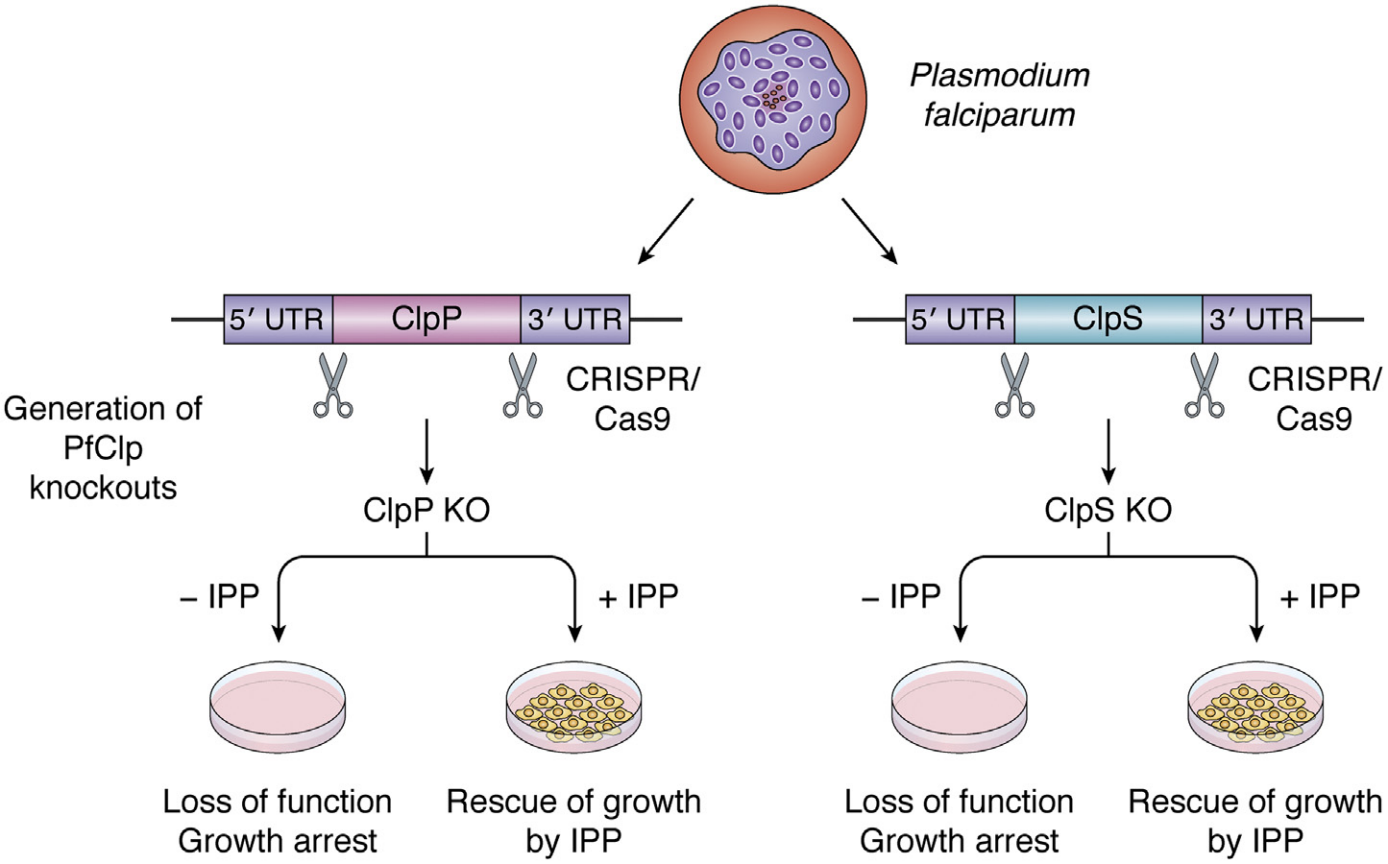
**Schematic representation summarizing the essential role of Clp components for apicoplast biogenesis and survival of *Plasmodium falciparum*.** ClpPKO and ClpSKO lines were generated by CRISPR-cas9. The absence of either ClpP or ClpS proteins led to the loss of function and growth arrest, suggesting that ClpP and ClpS are essential for apicoplast biogenesis and survival of the parasite. This growth inhibition was completely rescued by addition of the isoprenoid precursor IPP (isopentenyl pyrophosphate) (53). Clp, caseinolytic protease.

*T. gondii* and *P. falciparum* are both well studied members of the apicomplexan, but the apicoplast Clp system has only been studied in *P. falciparum*. *P. falciparum* apicoplasts contain single ClpP, ClpR, ClpC, and ClpS proteins (53). Functional apicoplasts are essential for the viability of the *P. falciparum* because apicoplasts synthetize and supply the cell with the isoprenoid precursor, isopentenyl pyrophosphate (IPP). Indeed, apicoplasts are dispensable for growth and viability of the organism if IPP is supplemented in the culture medium. A recent study reported on the engineering of a *P. falciparum* strain with a cytosolic mevalonate bypass system that produces isoprenoid precursors and therefore replicated normally after the loss of the apicoplast organelle (54). This approach provides an alternative to chemical complementation with IPP to probe the essential apicoplast function. ClpP, ClpR, ClpC, and surprisingly also ClpS (ClpS is not essential for viability in plants, algae, cyanobacteria, and nonphotosynthetic bacteria) are each essential for apicoplast viability because they are required for apicoplast biogenesis, and the absence of Clp components led to growth arrest and apicoplast loss (53, 55) (Fig. 3). Loss-of-function mutants for ClpP, ClpC, and ClpS could be obtained when grown in presence of IPP, allowing the introduction of modified ClpP and ClpC transgenes to probe Clp processing, assembly, protein–protein interactions, and trap substrates (53, 55). ClpP and ClpR form each homoheptameric rings, but they do not appear to form a stable tetradecameric complex together (56, 57). However, *in vivo* affinity tagging with ClpC, ClpP, or ClpS as bait, identified ClpR in all screens, suggesting that ClpR does form a complex with the rest of the Clp subunits. It has been observed for *E. coli* and plastid Clp protease core complexes that the intraheptameric ring interactions are far stronger than the interactions between the heptameric rings: an increase in ionic strength is sufficient to destabilize these ring–ring interactions, but not intraring interactions (58). This may explain why it has been difficult to purify an apicoplast ClpPR complex. It seems therefore quite likely that homoheptameric rings of apicoplast ClpR and ClpP do interact to form a complex. The structure of *P. falciparum* ClpR was determined by X-ray crystallography and showed that the ClpR monomer adopts a similar fold as bacterial ClpPs (57). *In vivo* experiments using *P. falciparum* expressing a catalytically inactive ClpP subunit and its endogenous functional ClpP subunit showed that ClpP undergoes processing by neighboring ClpP subunits to remove its prodomain. In contrast, ClpR has no prodomain and hence does not require any processing after its N-terminal signal peptide is cleaved (53). The structure of ClpS was determined by X-ray crystallography (59) and interaction with N-degrons investigated (59, 60), as will be discussed in more detail below (ClpS homologs and N-degrons across cyanobacteria, plastids, and apicoplasts).

## Discovery of Clp substrates and functions based on phenotypes, comparative proteomics, and *in vivo* and *in vitro* affinity enrichment

Clp adaptors and chaperones must selectively recognize the degradation signal in substrates through their substrate-interacting domains and deliver the substrates to the proteolytic chamber (21). Confirmed and candidate Clp substrates have been identified in cyanobacteria, chloroplasts, and apicoplasts by (i) a combination of comparative proteomics and follow-up studies, as reviewed (20, 21), (ii) discovery through direct interaction assays with adaptors ClpS1 and/or ClpF (43, 61) or NblA (62), or (iii) *in vivo* (*in planta*) trapping with the plastid ClpC chaperone (29) or ClpP, ClpR, ClpC, and ClpS proteins in apicoplasts (53), or (iv) targeted analysis of specific proteins to probe for the role of Clp instability, for example, for UmuD in cyanobacteria (42).

So far, no comparative proteomic screens or *in vivo* trapping approaches (as will be explained below) have been conducted for loss-of-function Clp mutants in cyanobacteria. It would be of great interest to carry out such studies for different cyanobacterial species. However, as mentioned above, there are two confirmed cyanobacterial Clp substrates, namely UmuD involved in DNA damage repair (42) and PBS proteins such as β-phycocyanin (62). The proteolysis adaptor, NblA, binds to the N-terminus of β-phycocyanin and delivers it to the ClpC chaperone. It should be noted that NblA is also involved in the disassembly of the protein pigment complex (62, 63, 64). Proteolytic ClpP1P2 complex activity is needed to support a normal circadian rhythm. It was suggested that the core oscillator protein KaiC is a target for the ClpXP protease because the KaiC level increased in the *clpxp2* null background under specific conditions (36). In the same study, it has also been proposed that a factor, defined as resetting factor, is targeted for degradation by the ClpXP1P2 protease system. It is important to note that *in vitro*, the Kai-based oscillator can function without the need for protein synthesis and degradation and relying on reversible phosphorylation and protein–protein interaction, but the *in vivo* scenario is more complicated, as discussed (36). Finally, no Clp protease activity besides ClpX function is needed to control cell division (even if the cell division protein FtsZ is a substrate of ClpXP in *E. coli* (65)).

The impact of the loss of chloroplast Clp capacity on plant phenotypes, through reduced accumulation of ClpC/D chaperones, ClpPRT subunits, or ClpS1 and ClpF adaptors, has been studied in *Arabidopsis* and to a lesser degree also in maize, tomato, rice, tobacco, and *Chlamydomonas*. Collectively, these studies suggest that Clp degrades a wide range of substrates and molecular plastid functions and that insufficient Clp protease or chaperone activity results in protein folding stress and probably protein aggregation (Clp in the proteostasis network, retrograde signaling and the cpUPR). Importantly, this has shown that the Clp system in *Arabidopsis* targets specific metabolic enzymes to help fine-tune metabolic activity, as exemplified by GluTR, a key enzyme in the tetrapyrrole pathway, deoxyxylulose 5-phosphate synthase (DXS) in the methylerythritol 4-phosphate pathway, phytoene synthase in the carotenoid pathway, and the thylakoid P-type ATPase of *Arabidopsis* 2 (PAAM2/HMA8) involved in copper transport, as summarized in an excellent review on control of plastid metabolism by the Clp system (20). Reduced activity of the Clp system also strongly affects plastid mRNA metabolism and plastid ribosomes, but it is still not clear whether these are pleiotropic effects or because the Clp system is essential to degrade or cleave specific proteins involved in plastid gene expression. Finally, other studies show the impact of the Clp system on tomato fruit ripening (66), and temporal proteomics using inducible RNAi lines of Clp subunits in *Nicotiana benthamiana* showed a very similar impact on the chloroplast proteome as in *Arabidopsis*, suggesting a conserved role of the Clp system across plant species (67). Two recent studies (68, 69) examined various phenotypic effects of the cosuppression of ClpC1 and ClpC2 in *N. benthamiana*. Similar as in *Arabidopsis*, loss of ClpC capacity resulted in reduced growth, virescent leaves, and a range of developmental defects. Not surprisingly, steady-state concentrations of a range of soluble primary metabolites were affected. These results underscore the essential nature of the Clp system not only in *Arabidopsis* but also in other plant species such as tobacco.

**Figure 4:**
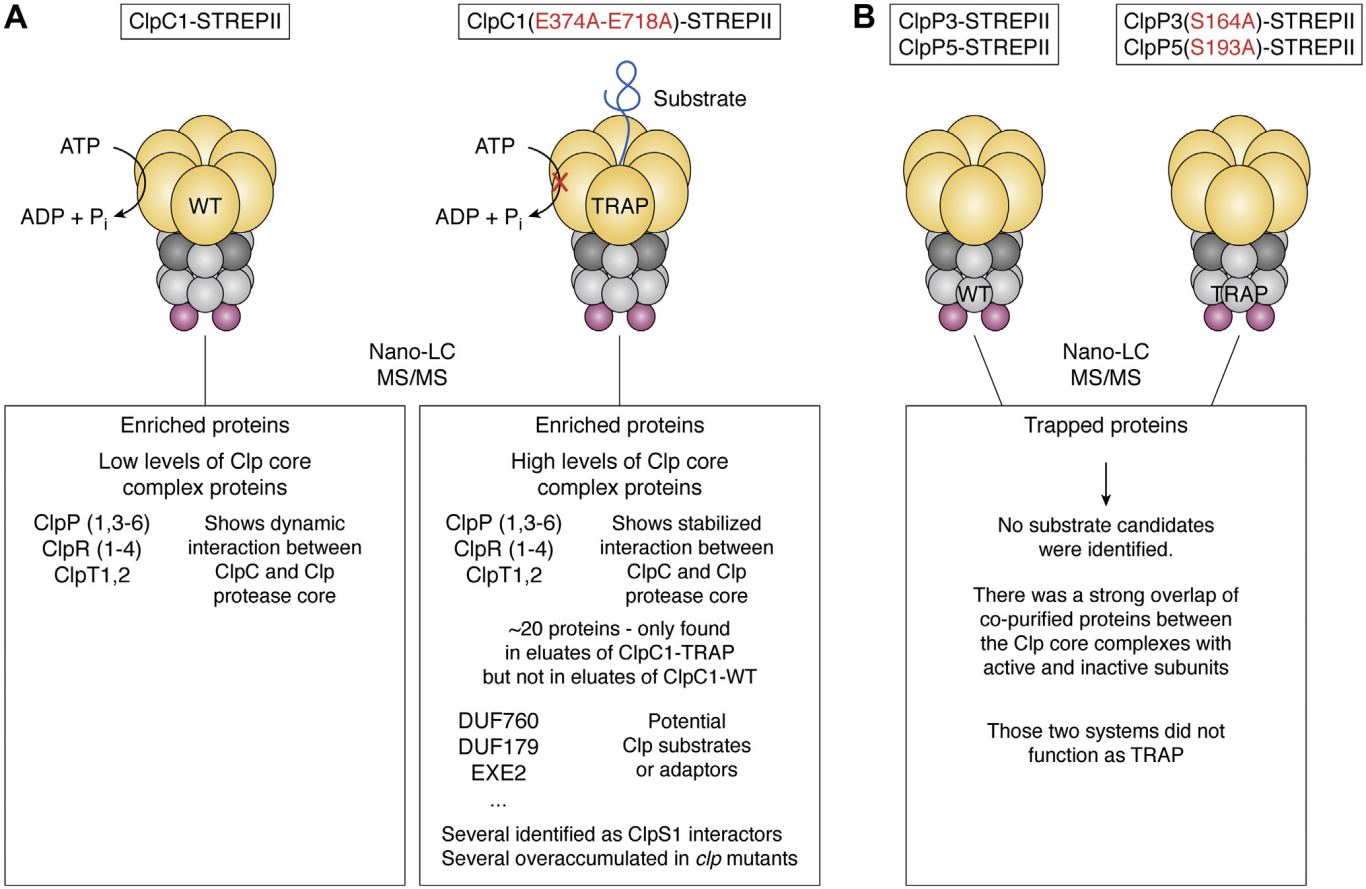
**Schema summarizing *in vivo* trapping in *Arabidopsis*.***A*, the *in vivo* trap was generated by expressing ClpC1 mutated in two critical glutamate residues in the two Walker B domains of ClpC1 required for the hydrolysis of ATP and with a C-terminal STREPII affinity tag for purification (ClpC1(E374A-E718A)-STREPII). ClpP (1, 3, 4, 5, 6), ClpR (1, 2, 3, 4) and ClpT1,2 subunits were strongly enriched in the ClpC1–TRAP, providing the first robust support for ClpC and Clp protease physical and functional interaction. A dozen potential substrates were only detected in eluates of ClpC1–TRAP but not in eluates of ClpC1-WT. Several of these trapped proteins also overaccumulated in Clp mutants and were previously identified as ClpS1 interactors, supporting the role of Clp proteases in the degradation of these targets (29). *B*, *in vivo* traps were generated by expressing ClpP3 or ClpP5 mutated in one critical serine residue and with a C-terminal STREPII affinity tag for purification (ClpP3(S164A)-STREPII and ClpP5(S193A)-STREPII). Those two systems did not function well as a TRAP compared with the ClpC1 TRAP. No substrate candidates were identified, suggesting that the bottle neck for degradation is likely substrate recognition and unfolding by Clp adaptors and chaperones, upstream of the Clp core (45).

An *in vivo* trapping approach in *Arabidopsis* identified a dozen potential substrates interacting with *Arabidopsis* chloroplast ClpC1 (29), following strategies successfully used for substrates trapping of other AAA+ proteins in bacterial systems, as reviewed (11). The *in vivo* trap was generated by expressing ClpC1 mutated in two critical glutamate residues in the two Walker B domains of ClpC1 required for the hydrolysis of ATP and with a C-terminal STREPII affinity tag for purification (29) (Fig. 4). When expressed in the WT, this ClpC1–TRAP induced a dominant visible phenotype, and no viable mutants that express ClpC1–TRAP in the *clpc1-1* null mutant could be recovered. This complete loss of viability and the dominant negative phenotype, together with MS evidence, suggests that (i) the ClpC1–TRAP forms inactive mixed oligomers with endogenous ClpC1 (in the WT background) and ClpC2 (in both WT and *clpc1-1* backgrounds) and (ii) the ClpC1–TRAP recruits and stably associates with Clp protease cores forming nonfunctional (jammed) complexes. Affinity purification of the ClpC1–TRAP followed by MS analysis resulted in a dozen proteins highly enriched compared with affinity-purified ClpC1 with a C-terminal STREPII affinity tag. These enriched proteins likely represent Clp protease substrates and/or new adaptors (Fig. 4). Several of these trapped proteins overaccumulated in *clp* mutants and/or were found as interactions for the adaptor ClpS1, supporting their functional relationship to Clp. Importantly, all ClpP (1, 3, 4, 5, 6), ClpR (1, 2, 3, 4), and ClpT1,2 subunits that make up the plastid protease core complex were strongly enriched in the ClpC1–TRAP complexes, providing the first robust support for ClpC and Clp core physical and functional interactions (29). *In vivo* traps were generated by expressing ClpP3 or ClpP5 mutated in one critical serine residue and with a C-terminal STREPII affinity tag for purification (ClpP3(S164A)-STREPII, ClpP5(S193A)-STREPII). Those two systems did not function well as a TRAP compared with the ClpC1 TRAP. No substrate candidates were identified, suggesting that the bottle neck for degradation is likely substrate recognition and unfolding by Clp adaptors and chaperones, upstream of the Clp core (45) (Fig. 3).

*In vivo* Clp protein interactome studies for the apicoplast Clp system in *P. falciparum* were carried out through affinity enrichments with ClpC, ClpR, ClpP, ClpP-inactive, and ClpS followed by MS to identify the enriched proteins (53) (Fig. 3). Enriched proteins were found for each of these affinity analyses. This supported the interaction between the four Clp subunits and identified multiple candidate substrates. Several proteins were identified in all five pull-downs and showed a diverse array of functions (*e.g.*, DNA replication, protein trafficking, RNA methyltransferase), whereas others were identified in four or less pull-downs. The pool of candidate Clp substrates is an excellent resource to probe apicoplast Clp function in more detail and discover degrons for the Clp system. We compared these Clp interactors with proteins identified in the *in vivo* ClpC1 trap for *Arabidopsis* (29) and confirmed plastid substrates, but no obvious overlapping homologs were identified.

## Clp in the proteostasis network, retrograde signaling, and the cpUPR

Cyanobacteria, plastids, and apicoplasts all contain multiple protease systems that collectively control protein processing and degradation. Based on the strong phenotypes of *clp* mutants with reduced Clp capacity, the Clp system clearly plays a central role in proteostasis in all three systems. Loss of Clp capacity not only affects the accumulation of specific proteins, as discussed in the previous sections, but also can result in protein folding stress and protein aggregation, leading to loss of viability, often referred to as proteotoxicity (70, 71). A typical response to proteotoxicity is the upregulation of chaperone capacity in an effort by the cell (or subcellular compartment) to remove protein aggregates and avoid more misfolding. Such upregulation involves signaling from the affected subcellular compartment to the nucleus to activate transcription of chaperones and possibly proteases and/or protein repair enzymes, summarized under the term unfolded protein response (UPR) (70, 71, 72, 73).

To the best of our knowledge, UPR pathways in response to loss of Clp capacity have not been studied in cyanobacteria. However, upregulation of chaperones in cyanobacteria is clearly associated with a variety of stresses including heat, cold, and reactive oxygen species stress (74, 75). A UPR signaling pathway has not been identified for apicoplasts, but it seems likely that such a pathway must exist to alleviate apicoplast proteome folding stress and aggregation. It will be important to evaluate a possible UPR to a loss of Clp capacity in cyanobacteria and apicoplasts and compare this to recent evidence for plastid UPR pathways in plants and algae.

Loss of chloroplast Clp capacity results in an increase of plastid chaperones CPN60/HSP70 and the unfoldase ClpB3 (52) as first observed in comparative proteomics of the *Arabidopsis clpr2-1* mutant (with only ∼20% Clp protease capacity) (76) and subsequently demonstrated in many additional comparative proteomics studies in *Arabidopsis*, as reviewed (20, 21). The upregulation of these chaperones suggests a retrograde signaling pathway that relays proteotoxic/folding stress in the chloroplast to the nucleus (20, 21, 77). The unfoldase activity of Arabidopsis ClpB3 was recently studied using recombinant ClpB3. It was shown to form a hexameric complex in the presence of MgATP and display intrinsic ATPase activity across a wide range of pH and was resistant to heat. This *in vitro* unfoldase activity was observed in absence of HSP70 (or other chloroplast stromal chaperones) and supports the role of ClpB3 as unfoldase/disaggregase in chloroplasts (52) and complements an earlier *Arabidopsis in vivo* study which showed that ClpB3 is critical for plastid biogenesis and possibly heat stress tolerance (50). A recent study in *Arabidopsis* identified the transcription factor HsfA2 (AT2G26150) as a component of this retrograde signaling pathway (77). Expression of HsfA2 was independent of the pentapeptide repeat protein Genomes Uncoupled 1 (GUN1; AT2G31400), a central integrator of chloroplast to nucleus retrograde signaling pathways. Interestingly, whereas GUN1 mRNA levels and the translation rate remain high across development, the half-life of the GUN1 protein is very short (∼4 h) because of ClpC-dependent degradation, resulting in low GUN1 protein levels under nonstress conditions (78). Thus, GUN1 is a deliberately unstable protein and is likely a genuine substrate of the Clp protease system. There is a clear parallel to the Ethylene Response Factor VII family of transcription factors involved in oxygen signaling and the anoxia response, as reviewed (79). These Ethylene Response Factor VII transcription factors are constantly degraded by the Cys/Arg branch of the N-degron pathway involving an N-recognin E3 ligase and the proteasome; however, at low cellular oxygen concentrations, degradation slows down, allowing these transcription factors to accumulate and activate the anoxia response (79). Testing a series of truncated GUN1 variants for *in vivo* stability suggested that the pentapeptide repeat domains play a role in stabilization, perhaps indicating that they function as (part of) a degron (78). However, it remains to be determined how GUN1 is recognized as a substrate and how it can be stabilized: many scenarios can be envisioned including a conformational change that could expose a degron, specific PTMs at selected amino acid residues generating or removing a degron, protection of a degron through binding by other proteins (*e.g.*, an antiadaptor), or even dynamic regulation of substrates’ adaptors.

A chloroplast Protein Unfolding Response (cpUPR) and retrograde signaling pathway was also proposed for the green algae *Chlamydomonas reinhardtii* (80). Recently, a cytosolic kinase MARS1 was identified as a chloroplast retrograde signaling component in *Chlamydomonas* (81). Loss of MARS1 (Cre16.g692228) reduced the ability of cells to cope with excessive light stress, whereas overexpression of MARS1 improved the cellular response to light stress. Furthermore, mutation of the catalytic triad of the kinase impaired MARS1 function in this signaling pathway. MARS1 was identified in a suppressor screen of cpUPR activation based on the expression of VIPP2 and HSP22 in response to downregulation of plastid-encoded ClpP1. These reporters are quite different in their functions than the stromal chaperones ClpB3/HSP70 that are used as markers for folding stress and aggregation in the *Arabidopsis* cpUPR studies. A recent study showed that VIPP2 forms a complex with its homolog VIPP1 and HSP22 to alleviate membrane stress and protein membranes from oxidative damage and to aid in the removal of dysfunctional proteins (82). BLAST searches identified AT1G67890 and AT5G49470 as the closest *Arabidopsis* MARS1 homologs in plants. Both *Arabidopsis* homologs have a PYP-like sensor domain (IPR035965) and a protein kinase domain. PYP-like sensor domain proteins have been identified in a wide range of species and often act as oxygen or redox sensors (83). Extensive work for higher plant chloroplasts identified multiple retrograde signaling pathways involving, for example, apocarotenoids, oxylipins, or 3′-phosphoadenosine 5′-phosphosulfate (84, 85, 86, 87). It is likely that multiple retrograde signaling pathways also operate in green algae such as *Chlamydomonas* (88). To further test if the MARS1-dependent pathway is truly involved in chloroplast protein folding and aggregation stress (rather than oxidative stress), it would be beneficial to test the genetic interaction between the Clp system and MARS1 and monitor the accumulation of plastid chaperones such as ClpB3.

In addition to being recognized for their function in unfolding and delivering substrates for degradation, chloroplast ClpC homologs have also been reported to participate in chloroplast protein import (89, 90). This was suggested because (i) a small fraction of total chloroplast ClpC1 has consistently been identified in chloroplast inner envelope fractions (most is in the stroma), (ii) Clp mutants have reduced import efficiency, and (iii) ClpC homologs have an affinity to chloroplast transit peptides. ClpC has also been suggested to aid in import quality control, that is, removal of precursor proteins that maybe dysfunctional. The precise functions and mechanistic details of ClpC contributions at the chloroplast inner envelop are controversial, as debated in recent letters published in the *Plant Cell* (89, 90). Probably for historical reasons, the chloroplast import literature refers to the ClpC homologs often as HSP93-III (ClpC2) and HSP93-V (ClpC1), with the roman numerical referring to the *Arabidopsis* chromosome number. This does add to the confusion because this family of AAA+ chaperones is referred to in the broader literature as Clp chaperones.

## ClpS homologs and N-degrons across cyanobacteria, plastids, and apicoplasts

Very little is known about the degrons for the Clp systems in cyanobacteria, plastid, or apicoplasts. Recognizing and understanding degrons is key to unraveling the role of Clp systems in proteostasis. Even in the most studied Clp system (*i.e.*, *E. coli*), understanding of substrate selection for degradation by Clp is far from complete. It has been demonstrated that substrate selection by the *E. coli* ClpXP system can involve the SsrA tag and the SsrB adaptor for protein nascent chains stalled on ribosomes, or a variety of C-terminal tags (10). The recognin/adaptor ClpS recognizes substrates based on degrons in the N-terminal region for delivery to ClpAP (*e.g.*, in *E. coli*) or ClpCP (*e.g.*, in *Mycobacterium tuberculosis*) (91, 92). Hence, ClpS fulfills a similar function as N-recognin E3 ligases for substrate selection and degradation by the proteasome (*e.g.*, PRT1 and PRT6 in *Arabidopsis thaliana*). The ClpA/C/X chaperones also recognize disordered regions (10, 93) as substrates. Adding to the complexity of substrate recognition and selection is the notion that adaptor proteins not only bind and deliver substrates but also they can also modulate substrate selection of proteases, as exemplified by, for example, HspQ and ClpS, influencing the activity of both ClpAP and the Lon protease in *Salmonella enterica* (94). In this section, we review the current understanding of ClpS homologs found in cyanobacteria, plastids, and apicoplasts and insight in N-degrons.

### Cyanobacteria

**Figure 5:**
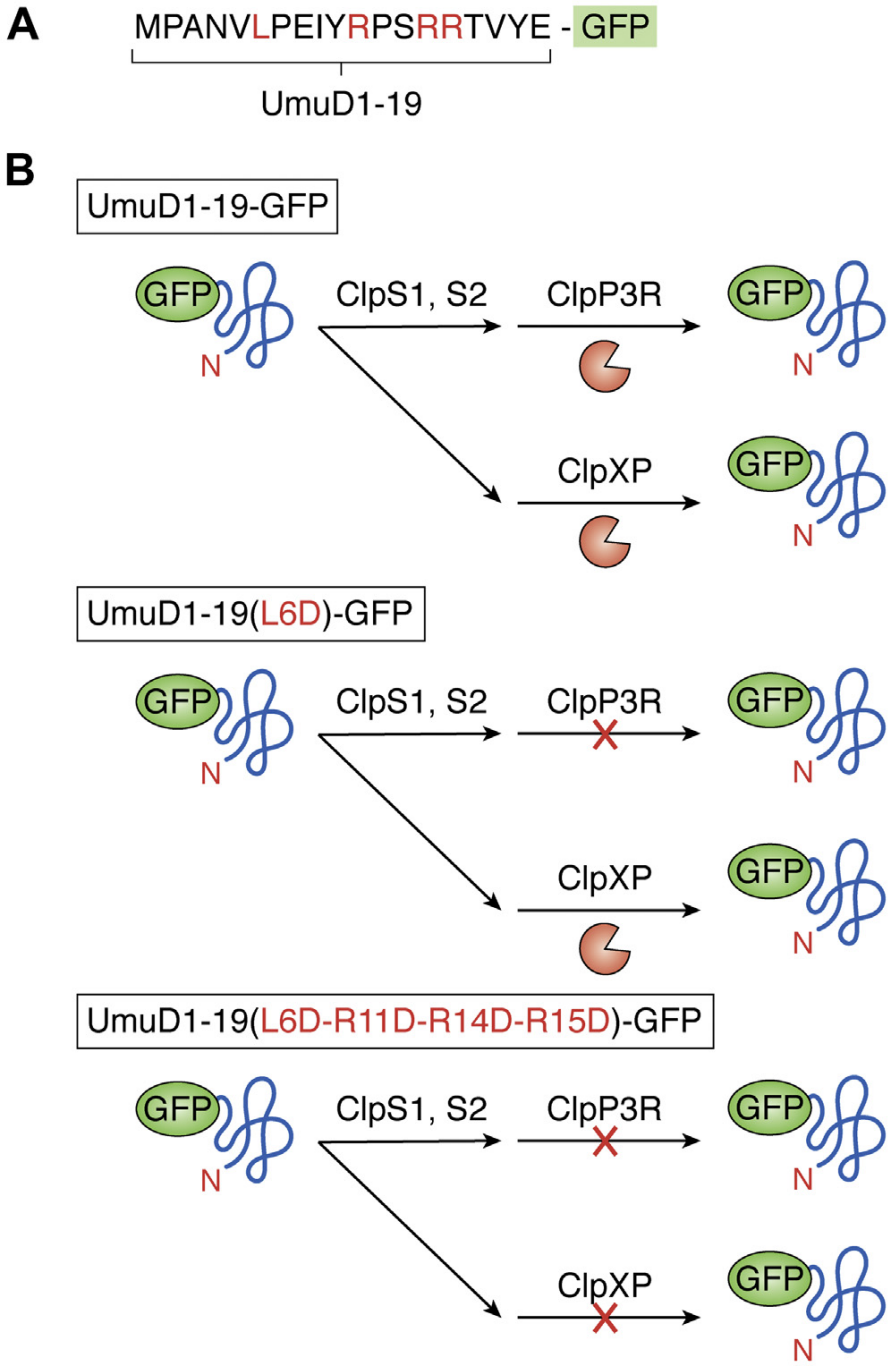
**Schematic representation of the dual degron of UmuD substrate of ClpCPR and ClpXP in cyanobacteria.***A*, amino acid sequence of the first 19 residues of UmuD in Synechocystis sp. PCC6803 (42). N-terminal region ((MPANVLPEIERPSRRTVYE) of UmuD protein (1–19 AA) fused to GFP (UmuD1-19AA-GFP). *B*, the N-terminus leucine residue (L6) is mutated to aspartate (D) (UmuD1-19AA-GFP(L6D)). The N-terminus leucine residue (L6) and the arginine residues (R11, R14, and R15) are mutated to aspartate (UmuD1-19AA-GFP (L6DR11DR14–15D)). L6 is essential for ClpS triggered degradation by ClpCPR and arginine residues in the N-terminal region contributed to degradation by ClpXP1P2 (42).

Not much is known about how Clp-driven proteolysis pathways are regulated in cyanobacteria. *Synechococcus elongates* contains ClpS1 and ClpS2 that dynamically associate with ClpC (95). Using peptide affinity arrays and recombinant ClpS1 and ClpS2, a differential affinity for N-terminal residues was observed (96). ClpS1 showed an affinity for F, Y, and W, whereas ClpS2 showed an affinity for L, F, and Y as well as V and I. In all cases, R or K at the P2 position (the residue immediately downstream of the N-terminal residue) increased binding, whereas D or E at the P2 position decreased binding (96). These *in vitro* results support ClpS-dependent N-degron pathway in cyanobacteria; however, N-degron pathways remain to be demonstrated *in vivo*. A recent breakthrough in the discovery of degrons in cyanobacteria came from a study in *Synechocystis* sp. *PCC6803* focused on *in vivo* degradation of UmuD involved in DNA damage repair. The UmuD homolog in *E. coli* was shown to be a substrate for both Clp and Lon, and degrons were located in the N-terminal region (97, 98) (Fig. 5). We note that, unlike *E. coli* and plastids in algae and plants, most members of cyanobacteria including, *Synechocystis* sp. *PCC6803*, lack Lon proteases (42). UmUD was degraded by ClpCP3R and required the presence of either ClpS1 or ClpS2, and in absence of both ClpS1 and ClpS2, UmUD was not degraded (42). Mutagenesis within the N-terminal region of UmuD (MPANVLPEIERPSRRTVYE) fused to GFP showed that the leucine residue was essential for ClpS-triggered degradation by ClpCP3R and that the arginine residues contributed to degradation by ClpXP1P2 (Fig. 5). *In vivo* N-degron substrates for *E. coli* ClpS were all found to be cleaved in the N-terminal region by unknown proteases, thus exposing the N-degron, and indicating that the ClpS-ClpAP pathway operated downstream of other proteases (99). A similar scenario may occur in *Synechocystis*, but despite significant efforts, the authors were unsuccessful to determine if leucine residue was the most N-terminal residue in the UmuD protein that ClpS recognized. Although this recent study (42) was a breakthrough in deciphering Clp degradation pathways in cyanobacteria, much remains to be done.

### Plastids

Phylogenetic analysis showed that green algae, moss, and angiosperms contain one or more copies of ClpS1 homologs but no ClpS2 homologs, and some species also contain ClpS1-like protein(s) (61, 100). Recently, we conducted *in vitro A. thaliana* ClpS1 affinity assays using eight N-degron protein reporters containing F, Y, L, W, I, or R in the N-terminal position and a positive residue (R) in the P2 position (101). Elution of the bound reporters with an FR dipeptide suggested that compared with *E. coli*, ClpS1 has a restricted N-degron specificity, recognizing proteins bearing an N-terminal F or W, only weakly recognizing L, but not recognizing Y or I. These findings could have been impacted by the choice of the dipeptide FR used to competitively elute the degron reporters. Our findings differed from a similar *in vitro* study (102), which suggested that *A. thaliana* ClpS1 affinity for F, Y, L, and W, with the lowest affinity for W. The 2.0 Å structure of *Arabidopsis* ClpS1 in the presence of a bound N-degron was recently determined by X-ray crystallography (103). Whereas many features of Arabidopsis ClpS1 are conserved, the structure showed a somewhat enlarged substrate binding pocket and reduced hydrophobic interactions with the ‘classical’ L N-degron. The hydrophobic residues in the pocket and the basic gatekeeper (R instead of M) at the entrance of the pocket control the N-degron selectivity of *Arabidopsis* ClpS1 (102, 103). *In vivo* testing of ClpS1 affinity for N-termini will be critical to truly assess ClpS1 substrate affinity and test the efficacy of ClpS delivery to ClpC. Large-scale *in vivo* chloroplast N-terminome analysis showed that A, V, T, and S, and to a lesser extent G and M, were the most frequent N-terminal residues of the steady-state stromal proteome, whereas other residues were very infrequent or absent as N-terminal residues. This suggested that N-degron pathways do operate in the stroma, but the rules remain to be determined (104). Moreover, we showed that *A. thaliana* ClpS1 interacts with ClpF, a protein unique to higher plant plastids (43). ClpF is likely to be a coadaptor of ClpS1, together involved in substrate (such as GluTR) delivery to the ClpC chaperones (22).

**Figure 6:**
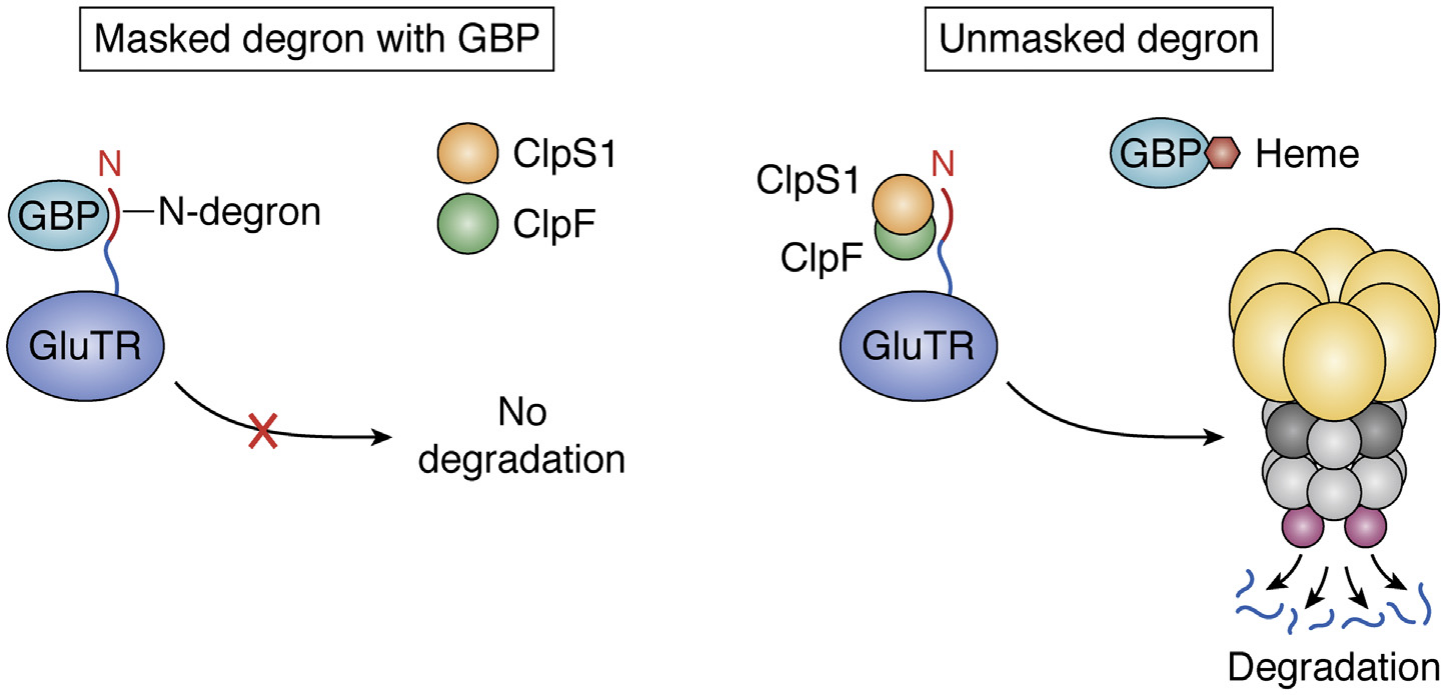
**Schema illustrating how GluTR N-degrons can be masked and unmasked by the GluTR-binding protein (GBP).** In the absence of heme, GBP binds to the N-degron of GluTR protein and prevents its degradation by the Clp system (masked N-degron). When heme levels increase, the release of GBP from GluTR1 enables the binding of ClpS1 and ClpF adaptors to the latter and its concomitant proteolytic degradation (unmasked N-degron) (106).

GluTR was identified as a direct interactor of ClpS1 using a combination of *in vitro* trapping and affinity purification with ClpS1 and mutated ClpS1 carrying a mutation in the ClpS1 substrate binding site (61). The interacting domains between ClpC1, ClpS1, ClpF, and GluTR were then mapped through *in vitro* biochemical studies using recombinant proteins, but there was no obvious degron in GluTR (43). Follow-up studies showed that GluTR degradation is delayed in Clp mutants and that expression of a truncated GluTR protein lacking the N-terminal region remained in the stroma (rather than associating with the thylakoid) and was more stable during prolonged darkness (105) (Fig. 6). Consistent with the role of the N-terminal domain as a degron, the rate of GluTR proteolysis in the dark increased in the absence of the GluTR-binding protein (GBP) but decreased in mutants impaired in ClpC1 or the proteolytic core subunit ClpR2 (105). Although GluTR accumulates at higher levels in ClpC1 and ClpS1 mutants, the degradation rate of the enzyme in the dark appears not to be altered when ClpS1 is missing. Accumulation of heme, induced by feeding with 5-aminolevulinic acid, stimulated Clp-protease–dependent degradation of *Arabidopsis* GluTR1. Binding of heme to the GBP inhibited the interaction of GBP with the N-terminal regulatory domain of GluTR1, thus making it accessible to the Clp protease (Fig. 6). These results show a functional link between the heme content and the post-translational control of GluTR stability by the Clp system, which helps ensure adequate availability of chlorophyll and heme (106).

### Apicoplasts

The structure of the apicoplast ClpS homolog has been determined by X-ray crystallography (59). *In vitro* ClpS affinity assays suggested that N-degrons included not only classic residues such as F, W, Y, L but also I (59, 60). Isoleucine is not a typical N-degron in other bacteria, which indicates that apicoplast ClpS exhibits an expanded substrate specificity. Despite this progress in identifying ClpS N-degrons *in vitro*, physiological substrates and *in vivo* demonstration of N-degron pathways apicoplasts have not yet been shown. The ClpS interactors identified using affinity purification screens discussed earlier (53) need to be further evaluated for their degrons and affinity to ClpS.

## Other substrate adaptors and delivery systems

Cyanobacteria contain PBSs that are large light-harvesting complexes that capture and funnel light energy to the photosystems. These complexes are degraded during nutrient deprivation leading to chlorosis (62). Degradation of PBS complexes is specifically triggered by proteolysis adaptors called nonbleaching proteins A and B (NblA and NblB) that act directly on the PBS (38, 62, 63, 107, 108). NblA is a small proteolysis adaptor of about 60 amino acids and its helix–loop–helix structure mimics fragments of the common PBP structure (41). This protein facilitates the interaction between ClpC and PBS. In fact, NblA interacts with PBS rods (109), α- and β-core subunits (41, 62), and ClpC (38). NblA can thereby induce degradation of both the PBS rod and core (39, 64). NblA expression is adjusted by a response regulator NblR and a sensor NblS (110). NblB is distantly related to the CpcE subunit of E/F-type lyases (108) and is implicated in the degradation of the core component allophycocyanin in the presence of NblA (63, 111).

In chloroplasts, HSP70 and the J20 cochaperone At4g13830 are involved as ClpC chaperone partners for the removal of aggregated and misfolded proteins (112, 113, 114, 115). In particular, the nonfunctional and aggregated forms of the chloroplast isoprenoid pathway enzyme (DXS) are delivered to Hsp70 by J20 that thus functions as an Hsp70 adaptor. HSP70 then delivers these nonfunctional DXS proteins to ClpC for degradation by the ClpPR protease or delivers them to the unfoldase ClpB3 for disaggregation (52). Indeed, mutants defective in J20 activity accumulated increased levels of DXS protein (but not transcripts) and displayed reduced levels of DXS enzyme activity, indicating that loss of J20 function causes post-transcriptional accumulation of DXS in an inactive form. This J20 protein is part of a larger family of J-domain containing proteins, also named J-proteins or DnaJ-like proteins, that can be divided in subfamilies based on the presence and absence of specific domains (116, 117). J-domain–containing proteins have been recognized as cochaperones for HSP proteins in bacteria (118), *Arabidopsis* (119, 120), yeast (121), and *Chlamydomonas* (122). Early on, J-domain proteins have also been recognized as important regulators for plastid biogenesis and light responses (123, 124, 125) and received new attention in recent years (116, 117). It is possible that in addition to the J20 protein At4g13830, other J-domain (like) proteins also could participate directly or indirectly in substrate recognition and delivery of plastid proteins for degradation.

Substrate adaptors have been identified for a variety of protease systems in both prokaryotes and eukaryotes. This includes E3-ligase adaptors and F-box proteins in the ubiquitin-proteasome degradation system in *Arabidopsis*, broadening the substrate selection capacity for cullin E3 ligases (126, 127, 128). The adaptors for these E3 ligases thus further enhance the capacity to select specific proteins for degradation. Similarly, diverse adaptors have been identified for Clp systems in bacteria such as RcdA, CpdR, and popA in *Caulobacter crescentus* or SsrB in *E coli*, as reviewed (35). We, therefore, speculate that, in addition to ClpS1 and ClpF, there could be additional adaptor proteins for the Clp system in chloroplasts. We suggest that there might be novel adaptors among the proteins specifically captured by the *in vivo* ClpC1 Walker B domain trap lines in *Arabidopsis* (29). However, further experimentation will be needed to test this hypothesis.

Finally, another mode to control protein degradation is through proteins that either directly protect substrates from targeting by adaptors or by proteins that modify the substrate selection by a protease. Both cases have been identified for the Clp system in nonphotosynthetic bacteria. For example, in *Salmonella enterica*, MgtC protects PhoP from degradation by the Clp system by outcompeting ClpS for binding to PhoP (129), whereas acetylated HspQ inhibits ClpS substrate selection by direct binding ClpS (94). Both MgtC and acetylated HspQ thus function as antiadaptors in the Clp system. It is quite striking that chloroplast ClpF also has an YccV domain similar as HspQ, perhaps suggesting that it can modify protease substrate selectivity and/or affect ClpS substrate selection. Future studies will be required to test these ClpF functions and antiadaptors and other protease specificity modulators in chloroplasts.

## Is the relationship between substrates and Clp conserved across lineages?

There are several specific cases of conserved substrates across diverse lineages, and these could be useful to explore to understand principles of substrate selection and degrons. Key examples are GluTR homologs involved in tetrapyrrole biosynthesis (for heme and chlorophyll) observed as Clp substrate in *Salmonella typhimurium* (130, 131) and chloroplasts, and UmuD involved in DNA repair in *E. coli* and cyanobacteria (42, 98). Systematic comparison across diverse lineages for these and other Clp substrates could help understand evolutionary adaptation in proteostasis.

## Conclusions and outlook

Clp systems are central in proteostasis in cyanobacteria, plastids, and apicoplasts. The basic components of these Clp machineries have now been established and several tools and techniques are available to better understand substrate degrons as well as recognition and delivery of substrates by known and unknown adaptors. In particular, *in vivo* substrate trapping techniques (11) under different abiotic conditions, developmental stages, and in loss-of-function mutants in genes that affect biogenesis or central plastid/proplastid functions, could provide a better understanding of the Clp substrate pool and the relevant degrons. Deliberate experimental and *in silico* strategies should be used to search and test for additional candidate Clp substrate adaptors.

Recent developments in CryoEM technologies and the establishment of many CryoEM facilities across universities and research institutions allow for structural determination of Clp complex assemblies, possibly together with stabilized and trapped substrates, as has been demonstrated in the last 2 years for several nonphotosynthetic bacterial systems (32, 33, 132, 133). Such structural information for plastid, apicoplast, and cyanobacterial Clp systems will be needed to truly understand mechanisms of substrate recognition, unfolding, and degradation.

Cyanobacteria, plastids, and apicoplasts require the activity of processing peptidases, proteases, and amino-peptidases (cleaving just one, two, or three amino acids) for a broad range of activities, including (i) removal of presequences of nuclear-encoded plastid and apicoplast proteins, (ii) N-terminal methionine removal of newly synthesized proteins, (iii) additional N- or C-terminal cleavages for maturation, stabilization, and possibly activation of proteins, (iv) removal of misfolded, damaged, or aggregated proteins, and (v) removal of unwanted proteins in response to environmental or developmental transitions. In particular, plastids are highly dynamic as they can undergo specialization during developmental transitions (*e.g.*, from etioplast to chloroplast, or from chloroplast to chromoplast). Therefore, coordination between the Clp protease systems and other proteases localized in the same subcellular compartments will be important and should be explored. A large-scale MS analysis of protein affinity enriched using a ClpS affinity column in *E. coli* suggested widespread proteolytic processing to generate N-end rule substrates for ClpS. Many of these affinity enriched substrates had also been modified by Aat (an amino transferase adding leucine or phenylalanine to proteins with N-terminal lysine or arginine) (99). This suggests that the N-degron pathway for the Clp system in *E. coli* operates mostly downstream of other proteases. Experimental tools such as comparative and quantitative peptidomics and proteomics in single and higher order protease mutant backgrounds are now sufficiently developed such that they can be effectively used to begin unraveling these protease networks. Finally, the adaptor ClpS (and possibly other adaptors) can be modified and tuned to deliberately target other proteins; this provides exciting opportunities for biotechnology and synthetic biology (134, 135).

## Conflict of interest

The authors declare that they have no conflicts of interest with the contents of this article.
